# Optimal blood pressure for patients with chronic kidney disease: a nationwide population-based cohort study

**DOI:** 10.1038/s41598-021-81328-y

**Published:** 2021-01-15

**Authors:** You-Bin Lee, Ji Sung Lee, So-hyeon Hong, Jung A. Kim, Eun Roh, Hye Jin Yoo, Sei Hyun Baik, Kyung Mook Choi

**Affiliations:** 1 Division of Endocrinology and Metabolism, Department of Internal Medicine, Korea University Guro Hospital, Korea University College of Medicine, 148 Gurodong-ro, Guro-gu, Seoul, 08308 Republic of Korea; 2 Division of Endocrinology and Metabolism, Department of Medicine, Samsung Medical Center, Sungkyunkwan University School of Medicine, Seoul, Republic of Korea; 3 Clinical Research Center, Asan Medical Center, College of Medicine, Ulsan University, Seoul, Republic of Korea

**Keywords:** Cardiology, Nephrology

## Abstract

The effect of blood pressure (BP) on the incident cardiovascular events, progression to end-stage renal disease (ESRD) and mortality were evaluated among chronic kidney disease (CKD) patients with and without antihypertensive treatment. This nationwide study used the Korean National Health Insurance Service-Health Screening Cohort data. The hazards of outcomes were analysed according to the systolic BP (SBP) or diastolic BP (DBP) among adults (aged ≥ 40 years) with CKD and without previous cardiovascular disease or ESRD (*n* = 22,278). The SBP and DBP were ≥ 130 mmHg and ≥ 80 mmHg in 10,809 (48.52%) and 11,583 (51.99%) participants, respectively. During a median 6.2 years, 1271 cardiovascular events, 201 ESRD incidents, and 1061 deaths were noted. Individuals with SBP ≥ 130 mmHg and DBP ≥ 80 mmHg had higher hazards of hypertension-related adverse outcomes compared to the references (SBP 120–129 mmHg and DBP 70–79 mmHg). SBP < 100 mmHg was associated with hazards of all-cause death, and composite of ESRD and all-cause death during follow-up only among the antihypertensive medication users suggesting that the BP should be < 130/80 mmHg and the SBP should not be < 100 mmHg with antihypertensive agents to prevent the adverse outcome risk of insufficient and excessive antihypertensive treatment in CKD patients.

## Introduction

Chronic kidney disease (CKD) is a major public health concern closely related to the risk of cardiovascular disease, progression to end-stage renal disease (ESRD), and premature mortality^1,2^. Hypertension is commonly accompanied by CKD and an important modifiable risk factor for cardiovascular events and CKD progression^3^. Therefore, controlling the blood pressure (BP) within an optimal range is a major public health priority and an important clinical concern for a patient with CKD.

Although reports including observational studies, clinical trials, and clinical practice guidelines, attempted to establish an optimal BP target among the CKD patients^4–13^, a definite consensus has not been achieved^14,15^ even in more recently released guidelines including the 2017 American Heart Association/American College of Cardiology (AHA/ACC)^12^ and the 2018 European Society of Cardiology/European Society of Hypertension (ESC/ESH)^13^ guidelines for managing hypertension. The Kidney Disease: Improving Global Outcomes (KDIGO) guideline recommends BP ≤ 140/90 mmHg for CKD patients with urine albumin excretion rate < 30 mg/24 h and ≤ 130/80 mmHg for CKD patients with moderately or severely increased albuminuria^5^. Although these recommendations have been accepted as reasonable, the supporting evidence was considered to be insufficient^5,15^. In contrast, the Eighth Joint National Committee on Prevention, Detection, Evaluation, and Treatment of High Blood Pressure (JNC 8)^10^ recommended BP < 140/90 mmHg for hypertensive adults with CKD, and the target BP did not vary according to the albuminuria status. In contrast, among individuals at high risk for cardiovascular events but without diabetes from the Systolic Blood Pressure Intervention Trial (SPRINT), targeting a systolic BP (SBP) < 120 mmHg, compared with < 140 mmHg, was associated with lower rates of major cardiovascular events and death from any cause, and no significant effect modification was observed according to the presence of CKD^11^. However, the lower limit of the BP target was not clearly defined. Furthermore, outcome trials or large-scale observational studies that evaluated an optimal BP target for CKD populations are extremely limited in Asia^16^.

Therefore, to identify the optimal BP in patients with CKD with respect to the risk of hypertension-related adverse outcomes, we aimed to (1) assess the distribution of BP levels and (2) compare the hazards of composite of progression to ESRD and all-cause death (primary outcome); all-cause death, and compositive of cardiovascular events and ESRD (secondary outcomes) during follow-up according to the baseline BP ranges among the individuals with CKD using the Korean National Health Insurance Service (KNHIS) database.

## Results

### Baseline characteristics of the study population

**Figure 1:**
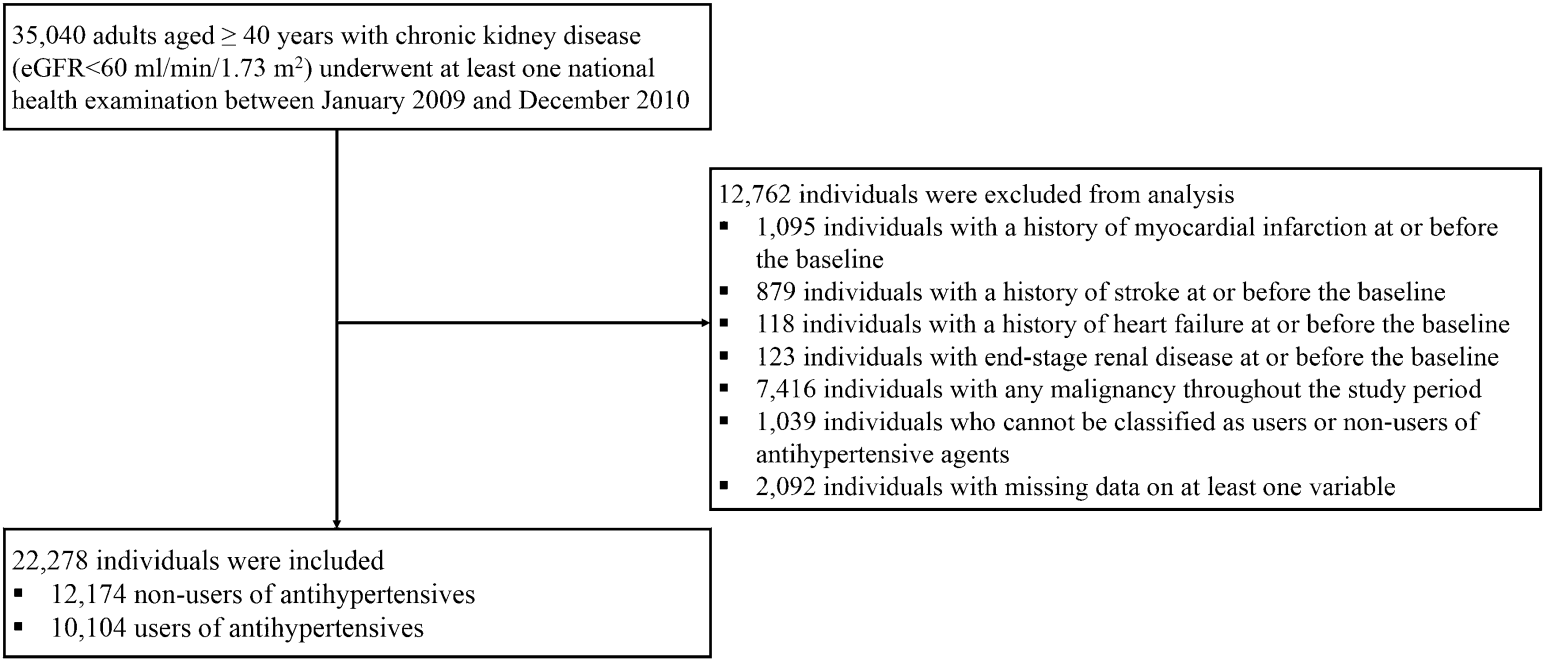
Flow diagram of the study population. Abbreviations: *eGFR* estimated glomerular filtration rate.

The study included 22,278 participants (Fig. 1). Among them, 12,174 were non-users and 10,104 were users of antihypertensive medications. The baseline characteristics of the total population and those of users and non-users of antihypertensive medications are summarised (Table 1). The mean age of the participants was 63.3 years, and 48.6% of these participants were men. The mean estimated glomerular filtration rate (eGFR) was 41.7 ml/min/1.73 m^2^ for the total population, and 5.9% of the population showed urine dipstick positivity for protein. The mean age, proportion of women, proportion of individuals with low-income status, prevalence of dyslipidaemia and diabetes mellitus (DM), and eGFR were higher among the users than among the non-users.

**Table 1:** Baseline characteristics of the study participants according to antihypertensive agent use.

	Total *n* = 22,278	Non-users *n* = 12,174	Users *n* = 10,104
Age (years)	63.3 ± 10.4	59.8 ± 10.0	67.4 ± 9.2
Men [*n* (%)]	10,836 (48.6)	6273 (51.5)	4563 (45.2)
Low-income status (lower 20%) [*n* (%)]	3008 (13.5)	1472 (12.1)	1536 (15.2)
Current smoker [*n* (%)]	2940 (13.2)	1926 (15.8)	1014 (10.0)
Heavy alcohol consumption [*n* (%)]	641 (2.9)	403 (3.3)	238 (2.4)
Regular exercise [*n* (%)]	4865 (21.8)	2764 (22.7)	2101 (20.8)
BMI (kg/m^2^)	24.3 ± 3.0	23.8 ± 2.8	24.9 ± 3.1
Waist circumference (cm)			
In men	85.5 ± 7.2	84.2 ± 6.8	87.3 ± 7.4
In women	80.8 ± 8.4	78.5 ± 7.8	83.2 ± 8.3
Systolic BP (mmHg)	127.2 ± 15.6	124.2 ± 14.9	130.9 ± 15.7
Diastolic BP (mmHg)	77.8 ± 10.0	76.9 ± 9.9	79.0 ± 10.0
Fasting plasma glucose (mg/dl)	104.0 ± 28.6	100.5 ± 24.5	108.3 ± 32.2
Total cholesterol (mg/dl)	202.6 ± 39.2	206.5 ± 37.9	198.0 ± 40.2
Triglyceride (mg/dl)	150.0 ± 94.1	144.4 ± 98.5	156.7 ± 88.1
HDL-C (mg/dl)	57.0 ± 50.6	60.2 ± 60.2	53.2 ± 35.5
LDL-C (mg/dl)	120.6 ± 37.0	124.6 ± 34.7	115.8 ± 39.0
Dyslipidaemia [*n* (%)]	10,913 (49.0)	4783 (39.3)	6130 (60.7)
Diabetes mellitus [*n* (%)]	5718 (25.7)	2096 (17.2)	3622 (35.8)
Proteinuria (urine dipstick positivity) [*n* (%)]	1320 (5.9)	425 (3.5)	895 (8.9)
eGFR (ml/min/1.73 m^2^)	41.7 ± 20.8	38.2 ± 23.3	45.9 ± 16.4
Categorization according to the baseline eGFR			
30 ≤ eGFR < 60 (ml/min/1.73 m^2^) [*n* (%)]	16,822 (75.5)	8118 (66.7)	8704 (86.1)
eGFR < 30 (ml/min/1.73 m^2^) [*n* (%)]	5456 (24.5)	4056 (33.3)	1400 (13.9)

*BMI* body mass index, *BP* blood pressure, *HDL-C* high-density lipoprotein cholesterol, *LDL-C* low-density lipoprotein cholesterol, *eGFR* estimated glomerular filtration rate.

Data are expressed as mean ± standard deviation (continuous variables) or n (%) (categorical variables).

Of the 22,278 (100%) participants, 4360 (19.6%) had an SBP ≥ 140 mmHg, and 10,809 (48.5%) had an SBP ≥ 130 mmHg. A total of 3016 (13.5%) participants had a diastolic BP (DBP) ≥ 90 mmHg, while 11,583 (52.0%) had a DBP ≥ 80 mmHg. Furthermore, of the 10,104 (100%) users of antihypertensive medications, 2703 (26.8%) had an SBP ≥ 140 mmHg, while 5920 (58.6%) had an SBP ≥ 130 mmHg. When the distribution of DBP was assessed among the antihypertensive users, the DBP was ≥ 90 mmHg in 1728 (17.1%) and ≥ 80 mmHg in 5792 (57.3%) users.

### Primary and secondary outcomes according to the categories of the SBP

During a median follow-up of 6.2 (interquartile range: 5.6–6.5) years, 1225 primary outcomes (the composite of ESRD and all-cause death) were observed. Individually, 1271 cardiovascular events, 201 incident ESRD cases, and 1061 all-cause deaths were noted. The hazard ratios (HRs) and 95% confidence intervals (CIs) for the primary and secondary outcomes are presented according to the prespecified SBP categories (Tables 2, and supplementary Table S2). When the SBP category of 120–129 mmHg was set as the reference, the SBP category of ≥ 140 mmHg was associated with higher hazards of the primary outcome (Table 2) and all-cause death (supplementary Table S2) during follow-up in the total population, and SBP category of ≥ 130 mmHg was associated with a higher hazard of composite of cardiovascular events and ESRD (supplementary Table S2) during follow-up in the total population. When stratified analyses according to the antihypertensive medication use were conducted, the individuals with SBP < 100 mmHg showed significantly higher hazards of the primary outcome (Table 2) and all-cause death (supplementary Table S2) during follow-up than those with SBP of 120–129 mmHg among the users of antihypertensive agents.

**Table 2:** Hazard ratios and 95% confidence intervals for the incidence of composite of end-stage renal disease and all-cause mortality according to the categories of systolic blood pressure among the individuals with chronic kidney disease.

Categories of SBP (mmHg)	Composite of end-stage renal disease and all-cause death			
	Events (n)	Follow-up duration (person-years)	Incidence rate (per 1000 person-years)	Adjusted HR (95% CI)*
**Total**				
< 100 (*n* = 389)	13	2337	5.56 (3.23–9.58)	0.936 (0.535–1.638)
100–109 (*n* = 1546)	51	9280	5.50 (4.18–7.23)	0.961 (0.708–1.303)
110–119 (*n* = 4285)	160	25,780	6.21 (5.32–7.25)	0.905 (0.739–1.109)
120–129 (*n* = 5249)	227	31,426	7.22 (6.34–8.23)	1 (Ref.)
130–139 (*n* = 6449)	357	38,610	9.25 (8.34–10.26)	1.126 (0.953–1.330)
140–149 (*n* = 2230)	191	12,989	14.70 (12.76–16.95)	**1.443 (1.190–1.751)**
≥ 150 (*n* = 2130)	226	12,374	18.26 (16.03–20.81)	**1.502 (1.247–1.811)**
*P* for trend				** < 0.0001**
**Non-users of antihypertensive agent**				
< 100 (*n* = 299)	0	1829	0	0.078 (0.005–1.257)
100–109 (*n* = 1139)	15	6929	2.16 (1.31–3.59)	0.679 (0.391–1.178)
110–119 (*n* = 2827)	57	17,193	3.32 (2.56–4.30)	0.889 (0.627–1.259)
120–129 (*n* = 3020)	71	18,344	3.87 (3.07–4.88)	1 (Ref.)
130–139 (*n* = 3232)	103	19,698	5.23 (4.31–6.34)	1.173 (0.866–1.587)
140–149 (*n* = 880)	45	5284	8.52 (6.36–11.41)	**1.489 (1.025–2.164)**
≥ 150 (*n* = 777)	54	4627	11.67 (8.94–15.24)	**1.744 (1.222–2.490)**
*P* for trend				** < 0.0001**
**Users of antihypertensive agent**				
< 100 (*n* = 90)	13	508	25.58 (14.86–44.06)	**2.023 (1.158–3.535)**
100–109 (*n* = 407)	36	2351	15.32 (11.05–21.23)	1.303 (0.908–1.871)
110–119 (*n* = 1458)	103	8587	12.00 (9.89–14.55)	0.948 (0.739–1.217)
120–129 (*n* = 2229)	156	13,082	11.92 (10.19–13.95)	1 (Ref.)
130–139 (*n* = 3217)	254	18,912	13.43 (11.88–15.19)	1.084 (0.888–1.324)
140–149 (*n* = 1350)	146	7705	18.95 (16.11–22.29)	**1.371 (1.093–1.720)**
≥ 150 (*n* = 1353)	172	7747	22.20 (19.12–25.78)	**1.376 (1.106–1.711)**
*P* for trend				**0.0022**

*SBP* systolic blood pressure, *HR* hazard ratio, *CI* confidence interval.

*Adjusted for age, sex, current smoking, alcohol consumption, regular exercise, household income level, body mass index, presence of dyslipidaemia, diabetes mellitus, and urine dipstick positivity for protein. Adjusted HRs (95% CIs) and P-values, considered to be statistically significant in bold font.

### Primary and secondary outcomes according to the DBP categories

The HRs (95% CIs) of primary and secondary outcomes were presented according to the prespecified DBP categories (Table 3, and supplementary Table S3). Compared with the DBP category of 70–79 mmHg (reference), the DBP category of ≥ 90 mmHg was associated with higher hazards of the primary outcome (composite of ESRD and all-cause death), and composite of cardiovascular events and ESRD during follow-up in the total population. The DBP category of ≥ 80 mmHg was associated with a higher hazard of all-cause death during follow-up than the reference range (DBP: 70–79 mmHg).

**Table 3:** Hazard ratios and 95% confidence intervals for the incidence of composite of end-stage renal disease and all-cause mortality according to the categories of diastolic blood pressure among the individuals with chronic kidney disease.

Categories of DBP (mmHg)	Composite of end-stage renal disease and all-cause death			
	Events (*n*)	Follow-up duration (person-years)	Incidence rate (per 1000 person-years)	Adjusted HR (95% CI)*
**Total**				
< 60 (*n* = 358)	26	2081	12.49 (8.51–18.35)	1.401 (0.940–2.088)
60–69 (*n* = 2964)	141	17,632	8.00 (6.78–9.43)	1.022 (0.840–1.243)
70–79 (*n* = 7373)	346	44,082	7.85 (7.06–8.72)	1 (Ref.)
80–89 (*n* = 8567)	452	51,341	8.80 (8.03–9.65)	1.150 (0.999–1.322)
90–99 (*n* = 2230)	184	13,040	14.11 (12.21–16.30)	**1.458 (1.218–1.746)**
≥ 100 (*n* = 786)	76	4619	16.45 (13.14–20.60)	**1.733 (1.350–2.224)**
*P* for trend				** < 0.0001**
**Non-users of antihypertensive agent**				
< 60 (*n* = 214)	6	1274	4.71 (2.12–10.48)	1.087 (0.478–2.471)
60–69 (*n* = 1863)	35	11,306	3.10 (2.22–4.31)	0.762 (0.522–1.113)
70–79 (*n* = 4306)	115	26,129	4.40 (3.67–5.28)	1 (Ref.)
80–89 (*n* = 4503)	122	27,468	4.44 (3.72–5.30)	1.008 (0.781–1.301)
90–99 (*n* = 927)	50	5536	9.03 (6.85–11.92)	**1.631 (1.169–2.275)**
≥ 100 (*n* = 361)	17	2190	7.76 (4.83–12.49)	1.451 (0.870–2.417)
*P* for trend				**0.0145**
**Users of antihypertensive agent**				
< 60 (*n* = 144)	20	807	24.79 (15.99–38.43)	1.477 (0.935–2.333)
60–69 (*n* = 1101)	106	6326	16.76 (13.85–20.27)	1.141 (0.906–1.437)
70–79 (*n* = 3067)	231	17,953	12.87 (11.31–14.64)	1 (Ref.)
80–89 (*n* = 4064)	330	23,873	13.82 (12.41–15.40)	**1.192 (1.007–1.410)**
90–99 (*n* = 1303)	134	7504	17.86 (15.08–21.15)	**1.345 (1.087–1.665)**
≥ 100 (*n* = 425)	59	2429	24.29 (18.82–31.35)	**1.825 (1.370–2.431)**
*P* for trend				**0.0042**

*DBP* diastolic blood pressure, *HR* hazard ratio, *CI* confidence interval.

*Adjusted for age, sex, current smoking, alcohol consumption, regular exercise, household income level, body mass index, presence of dyslipidaemia, diabetes mellitus, and urine dipstick positivity for protein. Adjusted HRs (95% CIs) and P-values, considered to be statistically significant in bold font.

## Discussion

In this nationwide population-based longitudinal study, including 22,278 participants with CKD, an SBP ≥ 130 mmHg was associated with significantly higher hazards of hypertension-related adverse outcomes compared with the reference range of 120–129 mmHg, while a DBP ≥ 80 mmHg was associated with higher hazards of hypertension-related adverse outcomes compared with the reference range of 70–79 mmHg. Nevertheless, approximately half of the participants exhibited an SBP ≥ 130 mmHg and DBP ≥ 80 mmHg. Among the antihypertensive medication users, an SBP < 100 mmHg was associated with a significant increase in the hazards of all-cause death, and composite of ESRD and all-cause death during follow-up.

To the best of our knowledge, this is the first study to evaluate the distribution of the SBP and DBP in a large-scale nationwide population with CKD in Asia. In this study, the SBP was ≥ 140 mmHg in 19.6% of the participants, and the DBP was ≥ 90 mmHg in 13.5% of the participants. When the distribution of the BP was evaluated among antihypertensive medication users only, 26.8% of the patients had an SBP ≥ 140 mmHg, while 17.1% had a DBP ≥ 90 mmHg. When the threshold BP was changed to 130/80 mmHg, almost half (48.5%) of the total participants showed an SBP ≥ 130 mmHg, and more than half (52.0%) exhibited a DBP ≥ 80 mmHg. The SBP was ≥ 130 mmHg in almost 60% of the antihypertensive medication users, and the DBP was ≥ 80 mmHg in a similar proportion of the antihypertensive medication users. These results suggest that the BP control status may be insufficient in a substantial proportion of the patients with CKD.

Over the past few decades, several studies attempted to address the optimal target ranges of the BP in the CKD population^4–11^. However, a definite consensus was not achieved, even among the clinical practice guidelines^5,9,10^. Recently, the SPRINT reported that an SBP target < 120 mmHg led to a decrease in the incidence of major cardiovascular events and death compared with an SBP < 140 mmHg among patients at high risk of cardiovascular events^11^. However, until recently, the extent to which the results from the SPRINT study should be applied to people with CKD, especially those with more progressed disease (eGFR < 45 ml/min/1.73 m^2^), is still under debate^17^. While the balance of risk and benefit of intensive BP control may differ across eGFR levels, the mean eGFR in the SPRINT participants with CKD was 48 ml/min/1.73 m^2^^11^, which was more favourable than that in our study (41.7 ml/min/1.73 m^2^). A post hoc analysis of the SPRINT study suggested an attenuation of the cardiovascular benefit from intensive treatment with lower eGFR, and no apparent net benefit was observed among 891 participants with an eGFR of < 45 ml/min/1.73 m^2^^18^. In our study, 24.5% of the participants had an eGFR < 30 ml/min/1.73 m^2^, and a BP ≥ 130/80 mmHg versus the reference range (120–129/70–79 mmHg) was associated with increased hazards of hypertension-related adverse outcomes, suggesting that the optimal BP range may be less than this level.

Here, an SBP < 100 mmHg was related to the hazards of all-cause death, and composite of ESRD and all-cause death during follow-up in patients treated with antihypertensive medications but not in individuals without antihypertensive therapy. Recently, Jung et al. reported different patterns of the association between the BP and adverse outcomes according to antihypertensive medication use in 492,540 Korean adults without pre-existing cardiorenal diseases^19^. In their study, the associations between the BP and adverse outcomes were linear or flat and then increasing among the non-users of antihypertensive agents but J-shaped among the active antihypertensive users. According to Jung et al.^19^, among the active users of antihypertensive agents, the risk for a composite of cardiovascular and renal outcomes increased in the individuals with SBP values < 115 mmHg and > 135 mmHg, and the risk for early all-cause death increased in those with SBP values < 125 mmHg and > 135 or 145 mmHg. Consistent with the results of a previous study, our study showed that the association of hazards for all-cause death, and composite of ESRD and all-cause death during follow-up with an SBP < 100 mmHg was found only among CKD patients using antihypertensive agents. These results suggest that an excessive BP lowering with antihypertensive medications may increase the risk of adverse outcomes also in individuals with CKD. The SBP cut-off was lower in the present study including only CKD patients (SBP < 100 mmHg) compared with that reported in a previous study including healthy individuals (SBP < 115–125 mmHg). Likewise, a J-shaped association of both the SBP and DBP with mortality has been reported for 651,749 U.S. veterans with CKD, and 53.7%, 49.7%, and 34.2% of them were taking renin–angiotensin–aldosterone system (RAS) inhibitors, beta-blockers, and calcium-channel blockers, respectively^8^. They reported that SBP of 140–160 mmHg and DBP of 80–90 mmHg were associated with best outcomes^8^ showing higher BP thresholds than our results. In their study^8^, patients with “ideal” BP (< 130/80 mmHg) demonstrated increased mortality rates possibly due to the inclusion of patients with low SBP and DBP. Another observational study including 2655 Japanese outpatients with CKD under nephrologist care showed that SBP < 110 mmHg and DBP < 70 mmHg increased risks of cardiovascular events and all-cause death^20^. However, these studies included only elderly veterans (mean age 73.8 years)^8^ or outpatients under nephrologist care from only 11 hospitals^20^. Moreover, both of these studies^8,20^ did not consider the possible differential impacts of BP on outcomes according to antihypertensive medication use.

There are several limitations of this study. First, although we tried to adjust for potential confounders, the effect of residual unmeasured confounders may remain. Second, BP measurement devices were heterogenous across institutions. However, quality assessments are applied to the BP measurement instruments in all health examination institutions according to the Basic Act on National Health Examination in Korea. Third, the present data did not include the ambulatory or home BP measurements. Fourth, categories of BP were defined based on BP measurements at a single timepoint. Repeated visit measurements and changes in BP during follow-up were not reflected due to the unavailability of the data in most of the participants. However, previous large epidemiological studies on the association between BP and adverse outcomes also utilized a single BP measurement to produce meaningful results^21–23^. In a study that compared statistical models that use simple summary measures of the repeat information on SBP (including baseline only and cumulative mean), against more complex methods that model the repeat information^24^, in comparison with the baseline-only model, modest benefits were observed using the cumulative mean of SBP, but any of the other complex methods demonstrated little further improvement. Fifth, CKD was defined only based on the eGFR; we could not reflect other features to characterise CKD, including albuminuria because except for the urine dipstick positivity for protein, the quantitative measures of albuminuria were not evaluated. Lastly, our study population consisted of Korean adults; thus, our results should be extrapolated to other ethnic groups with caution.

Our study has major strengths, including a comparatively large nationwide population across a broad age spectrum, high rates of participation, standardised data collection methods, an adequate follow-up period, and availability of comprehensive possible confounding factors, which support the validity of our results. Stratified analysis according to the antihypertensive medication use provided insights on the effect of BP lowering with antihypertensive agents and lower limit of BP targets. Our results may provide suggestions on the BP targets for Asian CKD patients, which have been lacking in relevant evidence.

This nationwide longitudinal population-based cohort study demonstrated that a BP ≥ 130/80 mmHg was associated with higher hazards of hypertension-related adverse outcomes compared with the reference range of 120–129/70–79 mmHg in adults with CKD. In approximately 50% of the Korean adults with CKD, SBP was ≥ 130 mmHg and DBP was ≥ 80 mmHg. Among the CKD patients using antihypertensive medications, those with an SBP < 100 mmHg showed higher hazards of all-cause death, and composite of ESRD and all-cause death during follow-up. These findings suggest that it would be appropriate to target the BP to < 130/80 mmHg and not to lower the SBP to < 100 mmHg with antihypertensive agents to prevent the risk of adverse outcomes associated with insufficient and excessive BP lowering among CKD patients aged ≥ 40 years. In this respect, the BP control status of Korean CKD patients need to be optimized. Further research is required to evaluate the optimal blood pressure target according to comorbidities and medications in patients with hypertension.

## Methods

### Data sources

The KNHIS-Health Screening Cohort (NHIS-HEALS) data from January 2002 to December 2015 were used in this study. Supplementary Appendix 1 provides details on the KNHIS and the NHIS-HEALS data. This study was approved by the Institutional Review Board (IRB) of Korea University (IRB file number: 2019GR0125). The requirement for obtaining informed consent was waived by the IRB of Korea University because the KNHIS provided the researchers with anonymized de-identified data. All methods were carried out in accordance with relevant guidelines and regulations.

### Study cohort, outcomes, and follow-up

In this nationwide longitudinal population-based cohort study, individuals aged ≥ 40 years with CKD at baseline who underwent ≥ one national health examination between January 2009 and December 2010 were included. CKD was defined as the eGFR < 60 ml/min/1.73 m^2^. The time-point of the examination conducted between 2009 and 2010 was considered as the baseline. The individuals with a history of myocardial infarction (MI) (ICD-10 codes I21–22), stroke (ICD-10 codes I63–64), heart failure (HF) (ICD-10 code I50), and/or ESRD at or before baseline, and those with malignancies (ICD-10 codes C00-C97) throughout the study period were excluded. The individuals who cannot be classified as users or non-users of antihypertensive medications and those with missing data for at least one variable were also excluded (Fig. 1).

The primary outcome was the composite of ESRD and all-cause death during follow-up. The secondary outcomes were all-cause death, and composite of cardiovascular events and ESRD during follow-up. The individuals with ESRD were defined as (1) those with one or more claims under ICD-10 codes N18–19 and who underwent haemodialysis (≥ 90 days), peritoneal dialysis (≥ 90 days), or kidney transplantation and/or (2) those who have had the health insurance benefit of relieved Co-payment Policy in Korea for ESRD, which is a welfare system in Korea aimed at reducing the related medical expenses for registered patients with certain severe illnesses, including ESRD^25^. Cardiovascular events were defined as a composite of newly diagnosed MI, stroke, and hospitalization for HF (hHF). Referring to the previous studies^23,26^, MI was identified as the recording of ICD-10 codes I21 or I22 during hospitalisation or claims made ≥ two times under those codes, while stroke was defined as the recording of ICD-10 codes I63 or I64 during hospitalisation with claims for brain magnetic resonance imaging or brain computed tomography. The hHF was defined as ≥ one hospitalisation under a primary diagnosis of ICD-10 code I50, as mentioned in the previous studies^27–29^. The study population was followed-up from baseline to the date of death, development of endpoint disease, or December 31, 2015, whichever came first.

### Measurements and definitions

Questionnaires were used to obtain information on the smoking status (never, past, or current), alcohol consumption, and regular exercise. Definitions of heavy alcohol consumption, regular exercise, low income status, and body mass index (BMI) are summarized in supplementary Table 1. eGFR was calculated using the Chronic Kidney Disease Epidemiology Collaboration equations^30,31^. Blood tests were performed using venous samples drawn after overnight fasting. These health examinations were conducted only in hospitals certified by the KNHIS. The presence of DM and dyslipidaemia was defined according to a previous study^26^.

During health examinations performed in hospitals certified by the KNHIS, the BP was measured by qualified medical personnel using sphygmomanometers or oscillometric devices at brachial levels after 5 min of rest in a sitting position.

The participants were stratified into two groups according to antihypertensive medication use^19^. The individuals who had been prescribed with antihypertensive medications for ≥ 180 days in the previous year from the baseline were categorised as users. Non-users were defined as those who (1) had been prescribed with antihypertensive medications for < 90 days in the previous year from the baseline and (2) had not been prescribed any antihypertensive medications in the past 6 months from the baseline.

### Statistical analyses

Statistical analyses were conducted using SAS software (Version 9·3, SAS Institute, Cary, NC, USA). Two-sided *p*-values < 0.05 were considered significant. The baseline characteristics of the study population were analysed in the total population and among users and non-users of antihypertensive agents. The continuous variables were expressed as mean ± standard deviation, whereas the categorical variables were presented as frequencies and percentages.

The incidence rates of outcomes were calculated as the number of incident cases divided by the follow-up duration (person-years). Multivariate Cox regression analyses were performed to calculate the HRs (95% CIs) for the outcome incidence according to the prespecified categories of baseline SBP or DBP. The prespecified SBP categories were < 100, 100–109, 110–119, 120–129, 130–139, 140–149, and ≥ 150 mmHg, while the DBP categories were < 60, 60–69, 70–79, 80–89, 90–99, and ≥ 100 mmHg. Regression models were adjusted for age, sex, current smoking, alcohol consumption, regular exercise, household income level, BMI, presence of dyslipidaemia, DM, and urine dipstick positivity for protein. Stratified analyses according to antihypertensive medication use were additionally performed.

### Data availability

The data that support the findings of this study are available from the Korean National Health Insurance Service (KNHIS) but restrictions apply to the availability of these data, which were used under license for the current study, and so are not publicly available. Data are however available from the authors upon reasonable request and with permission of the KNHIS.

## Supplementary Information

Supplementary Information.
