# Supplementary material for: Optimal blood pressure for patients with chronic kidney disease: a nationwide population-based cohort study
Source: Sci Rep. 2021 Jan 15;11:1538. doi: 10.1038/s41598-021-81328-y (PMC7810974; doi:10.1038/s41598-021-81328-y)
Supplement: Supplementary file 1 — Supplementary Information. [file 41598_2021_81328_MOESM1_ESM.docx]

**Supplementary Information**

**Article in *Scientific Reports***

**Optimal blood pressure for patients with chronic kidney disease: a nationwide population-based cohort study**

You-Bin Lee, Ji Sung Lee PhD^c,†^, So-hyeon Hong, MD^a^, Jung A Kim, MD^a^, Eun Roh, MD^a^, Hye Jin Yoo, MD^a^, Sei Hyun Baik, MD^a^, Kyung Mook Choi, MD^a,*^

Supplementary Appendix 1. Details on the Korean National Health Insurance Service (KNHIS) and KNHIS-Health Screening Cohort (NHIS-HEALS) data

Supplementary Table S1. Definitions of covariates

Supplementary Table S2. Hazard ratios and 95% confidence intervals for the incidence of composite of cardiovascular events and end-stage renal disease, and all-cause mortality according to the categories of systolic blood pressure among the individuals with chronic kidney disease

Supplementary Table S3. Hazard ratios and 95% confidence intervals for the incidence of composite of cardiovascular events and end-stage renal disease, and all-cause mortality according to the categories of diastolic blood pressure among the individuals with chronic kidney disease

**Supplementary Appendix 1.** **Details on the Korean National Health Insurance Service (KNHIS) and KNHIS-Health Screening Cohort (NHIS-HEALS) data**

As a single-insurer system, the KNHIS, operated by the Korean government, covers all the residents in Korea.^1^ Data regarding the anonymous identification numbers, demographic characteristics, monthly income, primary and secondary diagnoses classified according to the International Classification of Diseases-10th Revision (ICD-10), prescriptions, procedures, and dates of hospital visits and hospitalisations of all residents in Korea are compiled in the KNHIS database. The KNHIS actively operates a national health screening program among the Koreans.^1,2^ Through this program, standardised preventive health examinations are recommended at least every 2 years for the entire Korean adults aged ≥40 years.^1^ The data on smoking, alcohol consumption, physical activity, anthropometric measurements, such as height, weight, waist circumference (WC), and BP, and laboratory data, including estimated glomerular filtration rate (eGFR), urine dipstick test results, lipid profiles, and fasting plasma glucose level, are included in these standardised health examinations. The KNHIS constructed the NHIS-HEALS to provide researchers with representative population-based cohort datasets, including a substantial sample population of individuals who participated in health screening. This NHIS-HEALS cohort consists of 514,866 health screening participants accounting for 10% simple random sample of all individuals who participated in health screening in 2002–2003 and aged 40–79 years in 2002. The participants in this cohort were followed-up till December 2015 unless participants' eligibility was disqualified by the death or emigration.

**Appendix 1 References**

1. Seong SC, Kim YY, Park SK, Khang YH, Kim HC, Park JH *et al.* Cohort profile: the National Health Insurance Service-National Health Screening Cohort (NHIS-HEALS) in Korea. *BMJ Open* 2017;**7**:e016640.

2. Lee YH, Han K, Ko SH, Ko KS, Lee KU. Data Analytic Process of a Nationwide Population-Based Study Using National Health Information Database Established by National Health Insurance Service. *Diabetes Metab J* 2016;40:79-82.

**Table S1. Definitions of covariates**

| Variables | Definition |
| --- | --- |
| Heavy alcohol consumption | An average alcohol intake of ≥30 g/day |
| Regular exercise | Moderate-intensity physical activity accompanied by substantial shortness of breath for >30 minutes per session ≥5 days per week and/or high-intensity physical activity causing extreme shortness of breath for >20 minutes per session ≥3 days per week |
| Low-income status | Being among the lowest 20% of the population in the Korean National Health Insurance System, based on the monthly household income |
| Body mass index | Calculated as the body weight in kilograms divided by the height in meters squared (kg/m^2^) |

**Table S2. Hazard Ratios and 95% Confidence Intervals for the Incidence of Composite of Cardiovascular Events and End-Stage Renal Disease, and All-Cause Mortality According to the Categories of Systolic Blood Pressure Among the Individuals With Chronic Kidney Disease**

| Categories of SBP (mmHg) | **Composite of cardiovascular events and end-stage renal disease** | | | | **All-cause death** | | | |
| --- | --- | --- | --- | --- | --- | --- | --- | --- |
|  | Events (n) | Follow-up duration*^*^* | IR (per 1,000 person-years) | Adjusted HR (95% CI)^†^ | Events (n) | Follow-up duration*^*^* | IR (per 1,000 person-years) | Adjusted HR (95% CI)^†^ |
| **Total** | | | | | | | | |
| <100 (n=389) | 17 | 2,299 | 7.39 (4.60–11.89) | 1.156 (0.707–1.889) | 11 | 2,349 | 4.68 (2.59–8.46) | 0.888 (0.483–1.630) |
| 100–109 (n=1,546) | 59 | 9,156 | 6.44 (4.99–8.32) | 0.975 (0.734–1.294) | 44 | 9,304 | 4.73 (3.52–6.35) | 0.977 (0.705–1.356) |
| 110–119 (n=4,285) | 217 | 25,271 | 8.59 (7.52–9.81) | 1.099 (0.918–1.316) | 138 | 2,5842 | 5.34 (4.52–6.31) | 0.897 (0.722–1.115) |
| 120–129 (n=5,249) | 263 | 30,846 | 8.53 (7.56–9.62) | 1 (Ref.) | 201 | 31,521 | 6.38 (5.55–7.32) | 1 (Ref.) |
| 130–139 (n=6,449) | 429 | 37,623 | 11.40 (10.37–12.53) | **1.199 (1.028–1.398)** | 312 | 38,784 | 8.04 (7.20–8.99) | 1.100 (0.921–1.314) |
| 140–149 (n=2,230) | 188 | 12,591 | 14.93 (12.94–17.23) | **1.306 (1.082–1.577)** | 167 | 13,084 | 12.76 (10.97–14.85) | **1.388 (1.130–1.705)** |
| ≥150 (n=2,130) | 250 | 11,870 | 21.06 (18.61–23.84) | **1.562 (1.310–1.862)** | 188 | 12,529 | 15.01 (13.01–17.31) | **1.378 (1.127–1.685)** |
| *p* for trend |  | | | **<0.0001** |  | | | **<0.0001** |
| **Non–users of antihypertensive agent** | | | | | | | | |
| <100 (n=299) | 5 | 1,816 | 2.75 (1.15–6.61) | 0.775 (0.314–1.915) | 0 | 1,829 | 0 | 0.082 (0.005–1.342) |
| 100–109 (n=1,139) | 19 | 6,876 | 2.76 (1.76–4.33) | 0.772 (0.468–1.275) | 15 | 6,929 | 2.16 (1.31–3.59) | 0.737 (0.424–1.281) |
| 110–119 (n=2,827) | 73 | 17,012 | 4.29 (3.41–5.40) | 1.053 (0.766–1.449) | 54 | 17,201 | 3.14 (2.40–4.10) | 0.881 (0.617–1.258) |
| 120–129 (n=3,020) | 79 | 18,154 | 4.35 (3.49–5.43) | 1 (Ref.) | 69 | 18,353 | 3.76 (2.97–4.76) | 1 (Ref.) |
| 130–139 (n=3,232) | 124 | 19,400 | 6.39 (5.36–7.62) | **1.348 (1.017–1.788)** | 97 | 19,713 | 4.92 (4.03–6.00) | 1.101 (0.808–1.500) |
| 140–149 (n=880) | 52 | 5,146 | 10.11 (7.70–13.26) | **1.834 (1.291–2.605)** | 45 | 5,284 | 8.52 (6.36–11.41) | 1.415 (0.972–2.061) |
| ≥150 (n=777) | 59 | 4,500 | 13.11 (10.16–16.92) | **1.957 (1.393–2.749)** | 50 | 4,642 | 10.77 (8.16–14.21) | **1.505 (1.044–2.170)** |
| *p* for trend |  | | | **0.0002** |  | | | **0.0001** |
| **Users of antihypertensive agent** | | | | | | | | |
| <100 (n=90) | 12 | 483 | 24.84 (14.11–43.74) | 1.673 (0.933–3.001) | 11 | 520 | 21.16 (11.72–38.21) | **1.901 (1.038–3.482)** |
| 100–109 (n=407) | 40 | 2,280 | 17.55 (12.87–23.92) | 1.232 (0.875–1.734) | 29 | 2,375 | 12.21 (8.49–17.57) | 1.279 (0.856–1.909) |
| 110–119 (n=1,458) | 144 | 8,259 | 17.44 (14.81–20.53) | 1.174 (0.944–1.461) | 84 | 8,642 | 9.72 (7.85–12.04) | 0.929 (0.706–1.221) |
| 120–129 (n=2,229) | 184 | 12,692 | 14.50 (12.55–16.75) | 1 (Ref.) | 132 | 13,168 | 10.02 (8.45–11.89) | 1 (Ref.) |
| 130–139 (n=3,217) | 305 | 18,223 | 16.74 (14.96–18.72) | 1.110 (0.925–1.334) | 215 | 19,071 | 11.27 (9.86–12.89) | 1.085 (0.873–1.348) |
| 140–149 (n=1,350) | 136 | 7,445 | 18.27 (15.44–21.61) | 1.099 (0.880–1.373) | 122 | 7,800 | 15.64 (13.10–18.68) | **1.344 (1.050–1.721)** |
| ≥150 (n=1,353) | 191 | 7,370 | 25.91 (22.49–29.86) | **1.388 (1.132–1.702)** | 138 | 7,886 | 17.50 (14.81–20.68) | **1.301 (1.023–1.654)** |
| *p* for trend |  | | | 0.0950 |  | | | **0.0222** |

*^*^*In person-years.

^†^Adjusted for age, sex, current smoking, alcohol consumption, regular exercise, household income level, body mass index, presence of dyslipidaemia, diabetes mellitus, and urine dipstick positivity for protein.

SBP indicates systolic blood pressure; IR, incidence rate; HR, hazard ratio; CI, confidence interval.

**Table S3. Hazard ratios and 95% Confidence Intervals for the Incidence of Composite of Cardiovascular Events and End-Stage Renal Disease, and All-Cause Mortality According to the Categories of Diastolic Blood Pressure Among the Individuals With Chronic Kidney Disease**

| Categories of DBP (mmHg) | **Composite of cardiovascular events and end-stage renal disease** | | | | **All-cause death** | | | |
| --- | --- | --- | --- | --- | --- | --- | --- | --- |
|  | Events (n) | Follow-up duration*^*^* | IR (per 1,000 person-years) | Adjusted HR (95% CI)^†^ | Events  (n) | Follow-up duration*^*^* | IR (per 1,000 person-years) | Adjusted HR (95% CI)^†^ |
| **Total** | | | | | | | | |
| <60 (n=358) | 23 | 2,042 | 11.26 (7.49–16.95) | 1.081 (0.711–1.645) | 23 | 2,095 | 10.98 (7.29–16.52) | 1.396 (0.913–2.135) |
| 60–69 (n=2,964) | 167 | 17,283 | 9.66 (8.30–11.25) | 1.020 (0.852–1.220) | 117 | 17,716 | 6.60 (5.51–7.92) | 0.987 (0.797–1.223) |
| 70–79 (n=7,373) | 422 | 43,130 | 9.78 (8.89–10.76) | 1 (Ref.) | 298 | 44,262 | 6.73 (6.01–7.54) | 1 (Ref.) |
| 80–89 (n=8,567) | 546 | 50,089 | 10.90 (10.02–11.85) | 1.109 (0.977–1.260) | 401 | 51,538 | 7.78 (7.06–8.58) | **1.185 (1.020–1.377)** |
| 90–99 (n=2,230) | 184 | 12,660 | 14.53 (12.58–16.79) | **1.229 (1.033–1.463)** | 157 | 13,134 | 11.95 (10.22–13.98) | **1.470 (1.210–1.785)** |
| ≥100 (n=786) | 81 | 4,451 | 18.20 (14.64–22.63) | **1.456 (1.146–1.850)** | 65 | 4,667 | 13.93 (10.92–17.76) | **1.740 (1.329–2.278)** |
| *p* for trend |  | | | **0.0019** |  | | | **<0.0001** |
| **Non-users of antihypertensive agent** | | | | | | | | |
| <60 (n=214) | 6 | 1,259 | 4.76 (2.14–10.61) | 0.984 (0.434–2.229) | 6 | 1,274 | 4.71 (2.12–10.48) | 1.216 (0.549–2.691) |
| 60–69 (n=1,863) | 41 | 11,208 | 3.66 (2.69–4.97) | 0.749 (0.528–1.062) | 35 | 11,306 | 3.10 (2.22–4.31) | 0.811 (0.555–1.185) |
| 70–79 (n=4,306) | 138 | 25,788 | 5.35 (4.53–6.32) | 1 (Ref.) | 110 | 26,146 | 4.21 (3.49–5.07) | 1 (Ref.) |
| 80–89 (n=4,503) | 158 | 27,089 | 5.83 (4.99–6.82) | 1.070 (0.852–1.345) | 116 | 27,484 | 4.22 (3.52–5.06) | 0.993 (0.765–1.289) |
| 90–99 (n=927) | 47 | 5,419 | 8.67 (6.52–11.54) | 1.355 (0.972–1.887) | 47 | 5,548 | 8.47 (6.37–11.28) | **1.584 (1.126–2.230)** |
| ≥100 (n=361) | 21 | 2,140 | 9.81 (6.40–15.05) | 1.483 (0.936–2.349) | 16 | 2,194 | 7.29 (4.47–11.91) | 1.303 (0.775–2.193) |
| *p* for trend |  | | | **0.0214** |  | | | 0.0329 |
| **Users of antihypertensive agent** | | | | | | | | |
| <60 (n=144) | 17 | 783 | 21.72 (13.51–34.95) | 1.086 (0.665–1.772) | 17 | 821 | 20.71 (12.87–33.31) | 1.521 (0.930–2.486) |
| 60–69 (n=1,101) | 126 | 6,075 | 20.74 (17.42–24.70) | 1.155 (0.936–1.425) | 82 | 6,410 | 12.79 (10.30–15.88) | 1.094 (0.843–1.418) |
| 70–79 (n=3,067) | 284 | 17,342 | 16.38 (14.58–18.40) | 1 (Ref.) | 188 | 18,116 | 10.38 (9.00–11.97) | 1 (Ref.) |
| 80–89 (n=4,064) | 388 | 22,999 | 16.87 (15.27–18.63) | 1.101 (0.945–1.284) | 285 | 24,054 | 11.85 (10.55–13.31) | **1.275 (1.060–1.533)** |
| 90–99 (n=1,303) | 137 | 7,242 | 18.92 (16.00–22.37) | 1.129 (0.921–1.385) | 110 | 7,587 | 14.50 (12.03–17.48) | **1.403 (1.108–1.777)** |
| ≥100 (n=425) | 60 | 2,311 | 25.96 (20.16–33.44) | **1.431 (1.082–1.892)** | 49 | 2,474 | 19.81 (14.97–26.21) | **2.013 (1.469–2.758)** |
| *p* for trend |  | | | 0.2304 |  | | | **0.0004** |

*^*^*Adjusted for age, sex, current smoking, alcohol consumption, regular exercise, household income level, body mass index, presence of dyslipidaemia, diabetes mellitus, and urine dipstick positivity for protein.

DBP indicates diastolic blood pressure; IR, incidence rate; HR, hazard ratio; CI, confidence interval.
